# Selective Pro-Apoptotic Activity of Novel 3,3′-(Aryl/Alkyl-Methylene)Bis(2-Hydroxynaphthalene-1,4-Dione) Derivatives on Human Cancer Cells via the Induction Reactive Oxygen Species

**DOI:** 10.1371/journal.pone.0158694

**Published:** 2016-07-05

**Authors:** Pritam Sadhukhan, Sukanya Saha, Krishnendu Sinha, Goutam Brahmachari, Parames C. Sil

**Affiliations:** 1 Division of Molecular Medicine, Bose Institute, P-1/12, CIT Scheme VII M, Kolkata, 700054, India; 2 Laboratory of Natural Products & Organic Synthesis, Department of Chemistry, Visva-Bharati (a Central University), Santiniketan, 731235, West Bengal, India; National Institutes of Health, UNITED STATES

## Abstract

Selective induction of apoptosis in cancer cells barring the normal cells is considered as an effective strategy to combat cancer. In the present study, a series of twenty-two (22) synthetic 3,3'-(aryl/alkyl-methylene)bis(2-hydroxynaphthalene-1,4-dione) bis-lawsone derivatives were assayed for their pro-apoptotic activity in six different cell lines (five cancerous and one normal) using MTT assay. Out of these 22 test compounds, **1j** was found to be the most effective in inducing apoptosis in human glioma cells (CCF-4) among the different cell lines used in the study. The activity of this compound, **1j**, was then compared to a popular anticancer drug, cisplatin, having limited usage because of its nephrotoxic nature. In this study, **1j** derivative showed much less toxicity to the normal kidney cells compared to cisplatin, thus indicating the superiority of **1j** as a possible anticancer agent. This compound was observed to induce apoptosis in the glioma cells by inducing the caspase dependent apoptotic pathways via ROS and downregulating the PI3K/AKT/mTOR pathway. Estimation of different oxidative stress markers also confirms the induction of oxidative stress in 1j exposed cancer cells. The toxicity of **1j** compound toward cancer cells was confirmed further by different flow cytometrical analyses to estimate the mitochondrial membrane potential and cell cycle. The sensitivity of malignant cells to apoptosis, provoked by this synthetic derivative *in vitro*, deserves further studies in suitable *in vivo* models. These studies not only identified a novel anticancer drug candidate but also help to understand the metabolism of ROS and its application in cancer treatment.

## Introduction

Cancer is one of the leading causes of death in most of the countries. Cancer develops when somatic cells mutate and escape the restraints that normally restrict them from their problematic expansion [1–3]. Despite the presence of remarkably effective tumor-suppressing mechanisms that can discriminate between abnormally growing (neoplastic) and normal cellular states and competently suppress the former irrespective of the later, cancer develops. Different environmental conditions such as pollution, certain infections, radiation, etc. [4], and human habits, like the use of tobacco, are a few examples that increase the risk of cancer [5]. At the molecular level, a distinct difference lies in the redox metabolism of carcinomas and normal healthy tissues. The enhanced levels of intracellular reactive oxygen species (ROS) are usually observed in cancer cells [6,7]. Moreover, reductive features, like hypoxia and high metabolic activity are also reported to be associated with such tumor cells [8]. Thus, for cancer therapy, interfering with the redox homeostasis of these cancer cells appears as a promising approach. Based on this fact, numerous efforts have been made to design chemotherapeutic drugs. These molecules have shown to interfere with the redox balance within the cancer cells, specifically by targeting their altered redox conditions [9]. In addition, inhibitors of different growth factors involved in cancer signalling cascades, (*e*.*g*. RAS/ERK, PI3k/Akt, JAK/STAT, etc.) are promising in preventing cancers. Moreover, some genes that regulate apoptosis have been observed to be defective in malignant cells [10]. Recent research reports suggest that induction of apoptosis by modulating the expression of different apoptosis regulatory genes is very effective in mitigating cancer. Onset of apoptosis has substantial impact on the initiation, progression as well as metastasis of tumour [11,12].

In this study, we comprehensively analyzed the cytotoxicity of a series of synthetic bis-lawsone compounds. *Lawsonia inermis* Linn. (Lythraceae), also known as Henna or Mehndi, traditionally used all over the world as cosmetics and herbal remedies in treating various ailments [13], is a major natural source of lawsone (2-hydroxy-1,4-naphthoquinone). This chemical entity has been reported to exhibit a wide range of promising biological and pharmacological activities including antioxidant [14], antimicrobial [15,16], trypsin enzyme inhibition [17], anticoagulant [18] and antidiabetic [19,20]. Under this preview, one of our group members has recently synthesized a series of novel 3,3'-(aryl/alkyl-methylene)bis(2-hydroxynaphthalene-1,4-dione) scaffolds from the reaction of lawsone and different aldehydes following a novel protocol [21] with an intention that the synthetic bis-lawsone derivatives bearing lawsone as a sub-structure, may exhibit certain promising biological activities. Again, hydroxynapthalene [22] and arylmethylene [23,24] derivatives are reported to possess effective antimicrobial, herbicidal and antioxidant activities. Development of diverse hydroxynapthalene and arylmethylene scaffolds with anticancer activity could, thus, be expected to have clinical importance.

**Fig 1:**
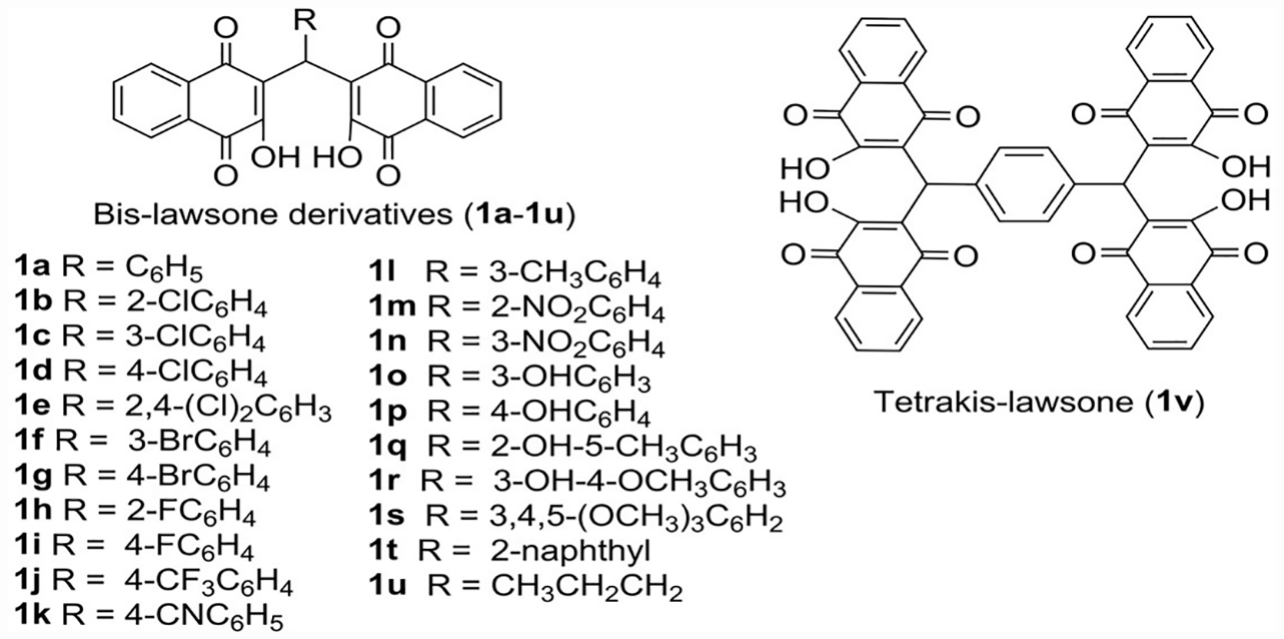
Chemical structures of the synthesized bis-lawsone compounds.

Most of the test compounds in the series of twenty-two bis-lawsone derivatives exhibited cytotoxicity to all types of cancer cells screened in our present study. Interestingly, few of these compounds were found to be non-toxic to the normal cells as well. Among the test compounds, **1j** [i.e. 3,3'-((4-(trifluoromethyl)phenyl)methylene)bis(2-hydroxynaphthalene-1,4-dione)], was observed prominently cytotoxic to the cancer cells but not to the normal cells. Compound **1j** contains a trifluoromethyl group (-CF_3_), a strong electron-withdrawing group, substituted at the 4-position of the phenyl ring (Fig 1). This kind of fluorinated moieties in heterocyclic compouds are belived to interfer with the lipophilicity, metabolic stability and bioavailability of the compund. This selected derivative (**1j**) was found to be the most cytotoxic to glioma cells and significantly non-toxic to the normal kidney cells. Later, we compared the proapoptotic activity of this **1j** derivative against a well-known anticancer drug, cisplatin or *cis*-diamminedichloroplatinum(II) (CDDP). Cisplatin is a very popular platinum-containing chemotherapeutic drug, clinically used to treat various types of cancers [25,26]. Currently used chemotherapeutic treatments are apparently effective but showing the severe side effects of these agents impose restrictions in their use. Besides, increasing evidence of the relapse of mammalian tumors are the limitations of most of the anticancer agents that are presently in use and CDDP is no exception to it. This provokes scientists around the globe to develop novel anti-cancer drugs with insignificant side effects. The present study was, therefore, designed to explore the cytotoxic effect of aforementioned napthaquinone derivatives and after the identification of the effective one(s) in the series, further experiments were also planned to determine the molecular pathways through which the identified derivative(s) exerts cytotoxicity in cancer cells. MTT cell viability assay was initially performed to assess the cytotoxic potentials of the 22 bislawsone derivatives and finally the study was continued with the most effective cytotoxic derivative, **1j**, to evaluate its mode of action. Wound healing and clonogenic assays were also performed to assess the anticancer property of this compound. Further, this study is expected to shed some light in understanding the role of oxidative stress in cancer biology.

## Materials and Methods

### Chemicals

RPMI-1640 media, DMEM media and other necessary chemicals like antibiotics, amino acids, etc. were purchased from HIMEDIA (Mumbai, India). Fetal bovine serum (FBS) was purchased from HyClone (Thermo Scientific Hy-Clone, Logan, Utah, USA). Methylthiazolyldiphenyl-tetrazolium bromide (MTT) was purchased from Sisco Research Laboratory (Mumbai, India). Cisplatin, ribonuclease, Fluorescein isothiocyanate (FITC) conjugated Annexin V, apoptosis detection kit, Z-vad FMK, RNaseA, Bradford reagent, luminol and coumaric acid were purchased from Sigma (Missouri, USA). Antibodies were purchased from abcam (Cambridge, UK) and Cell Signaling Technology (Danvers, MA, USA). Other essential chemicals used in this study were of the analytical grade.

### Bis-lawsone compounds

All the bis-lawsone derivatives (i.e. 3,3'-(aryl/alkyl-methylene)bis(2-hydroxynaphthalene-1,4-dione) derivatives; **1a**-**1u**) and one tetrakis-lawsone derivative 1v were synthesized from lawsone (i.e. 2-hydroxynaphthalene-1,4-dione) and aldehydes in aqueous ethanol at room temperature using commercially available sulfamic acid as an inexpensive and environmentally benign organo-catalyst following the methodology as reported earlier [21]. All the synthesized compounds were fully characterized based on their analytical and spectral studies (FT-IR, ^1^H NMR, ^13^C NMR and TOF-MS) and comparison of these data with those reported in the literature [21].

### Cell lines

In the present study, five cancerous cell lines [namely, human glioblastoma multiforme cell line (CCF-4), human kidney carcinoma epithelial cell line (A498), human cancerous lung epithelial cell line (A549), human cervix adenocarcinoma epithelial cell line (HeLa) and human renal cell carcinoma (RCC) line SK-RC-45] and one normal cell line [namely, human normal kidney epithelial cell line (NKE)] were used. A498, A549 and HeLa cell lines were obtained from NCCS, Pune. CCF-4, SK-RC- 45 and NKE cell lines were obtained as a gift from Dr. Kaushik Biswas, Bose Institute. The cells were cultured in a T-75 flask with RPMI-1640 (for A498, SK-RC-45, CCF-4, NKE) or DMEM (for HeLa, A549) supplemented with 10% FBS and antibiotics (100 U mL^−1^ of penicillin, 100 μg mL^−1^ of streptomycin, 50 μg mL^−1^ gentamicine and 2.5 μg mL^−1^ amphotericin B) at 37°C in a humidified incubator with 5% CO_2_.

### Drug Exposure

All the compounds were dissolved in DMSO and added to the cells at different concentrations. After the treatment with the desired compounds for specified time, the extent and nature of toxicity was determined. Finally, the toxicological assessment of CDDP and **1j** molecules on both CCF-4 and NKE cell lines was carried out with their respective LC_50_ values.

### Cell viability assay & determination of LC50

At first, the cells were seeded in flat-bottomed 96-well plates at a density of 0.1 × 10^6^ cells per well. For dose dependent study, the cells were exposed to 5, 10, 20 and 40 μM of the compounds for 24h. The optimum time of exposure and assay conditions were chosen appropriately based on the time dependent assay (data not shown). After the incubation period, the cell viability was measured by MTT assay following the method as described elsewhere [27,28]. The LC_50_ values for individual compounds were then determined. Later, this experiment was repeated with the most effective molecule (derivative) on the CCF-4 cells and the result was compared with another potent anticancer drug CDDP. The most effective compound was chosen on the basis of their effect on both the cancer cell lines and the normal cell line.

### LDH cytotoxicity assay

Lactate dehydrogenase (LDH) release from cells was determined using LDH assay kit to confirm cell damage [29]. The experiments were carried out following the manufacturer’s protocol (Lactate Dehydrogenase Activity Assay Kit (MAK066) manufactured by Sigma-Aldrich).

### Apoptosis detection and quantification

CCF-4 and NKE cells (80% confluency) were cultured in 6-well plates and incubated with **1j** in separate wells. After 24h treatment, the cells were scraped and centrifuged at room temperature (300 x g, 5 minutes). The cell pellets were washed with PBS, and were suspended in Annexin V Binding Buffer to which 1 μl of Annexin V/FITC was added and incubated for 5 minutes in dark (at room temperature). Immediately after incubation, the samples were analyzed flow cytometrically in FACS Verse with an excitation of 488 nm and emission of 520 nm [30].

### Determination of the intracellular ROS

This assay was done following the method of Cossarizza [31]. Briefly, the CCF-4 and NKE cells were incubated separately with the **1j** derivative and CDDP at their optimum toxic dose. Then after scrapping, cells were pelleted down by centrifugation (300 x g, 5 minutes, room temperature). After that, these pellets were dissolved in PBS and incubated with H_2_DCFDA (2 μM). Following incubation in the dark for 20 minutes at 37°C, the samples were analyzed by FACS Verse at excitation and emission wavelengths of 488 nm and 520 nm respectively [32]. In addition, intracellular ROS was measured using fluorescent microscope by following the method described elsewhere [28]. Briefly, both the cells were cultured overnight on coverslip, followed by the incubation with the 20 μM **1j** and 5mM NAC. After 24 h of incubation, cells were incubated with H_2_DCFDA as mentioned earlier. Then cells were washed with PBS and mounted with fluorescent medium in a glass slide and observed under a fluorescent microscope at 20X magnification.

### Estimation of the intracellular GSH and GSSG

The level of GSH was determined following the protocol described elsewhere using Ellman’s reagent [33–35]. Briefly, after treatment with desired compounds, the cells were sonicated and then centrifuged (12000 rpm, 15 minutes) for total protein isolation. DTNB solution (Ellman’s reagent) was added to the protein containing supernatant and spectrophotometrically the absorbance at 412 nm was measured. The method of Hissin and Hilf (1976) was used to measure the level of GSSG [36]. Samples were mixed with 0.04 M NEM to prevent the oxidation of GSH to GSSG and incubated for 30 minutes at room temperature. Then 300 mM Na_2_HPO_4_ and Ellman’s reagent were added. Finally, the absorbance was recorded at 420 nm.

### Estimation of lipid peroxidation

To ensure the occurrence of oxidative stress in the cancer cells, the extent of lipid peroxidation was determined by estimating the level of malondialdehyde (MDA) in the cells. Briefly, the control and treated cells from different experimental group was collected and subjected to sonication. Each of the sonicated samples were then mixed with 20% trichloro acetic acid and 0.67% thiobarbituric acid. After that, the mixtures were heated at 100°C for 1 hour and then kept in a ice bucket for cooling. Finally, the solutions were centrifuged and the absorbance was measured at 535 nm using a spectrophotometer [37].

### Assessment of antioxidant enzymes

The activities of the antioxidant enzymes, superoxide dismutase (SOD) and catalase (CAT) were measured using spectrophotometer following the protocol as described elsewhere [38].

### Determination of mitochondrial membrane potential

The mitochondrial membrane potential was determined following the method of Salvoli *et al* [39,40]. Following proper treatments for each set, cells were incubated separately with 5 mM JC-1 dye (at 37°C for 30 minutes) followed by centrifugation (5 minutes, 300 x g) and suspension in PBS. The fluorescence-labeled cells were analyzed flow-cytometrically at the excitation and emission wavelengths of 530 nm and 590 nm respectively by BD FACS Calibur Flow Cytometry System (BD Biosciences).

### Immunoblotting

The treated cells were washed with PBS and lysed using RIPA buffer containing 150 mM sodium chloride, Triton X-100, 0.5% sodium deoxycholate, 0.1% SDS (sodium dodecyl sulfate), 50 mM Tris and protease and phosphatase inhibitors at pH 8.0. The lysed cells were centrifuged at 12,000 rpm at 4°C for 10 minutes. The supernatant was collected, and protein content was measured using BCA method. The protein samples were stored at -80°C for further use. To perform the immunoblot analysis, 50 μg of protein from each sample was resolved by SDS-PAGE and then transferred to PVDF membranes. The membranes were blocked using 1% BSA in TBST buffer for two hours at room temperature. The membranes were then incubated overnight with respective primary antibody at 4°C. Next, the membranes were washed with wash buffer (50 mM/L Tris–HCl, pH 7.6, 150 mM/L NaCl, 0.1%Tween 20) and incubated with specific HRP-conjugated secondary antibody for 2 hours at room temperature. Finally, the membranes were developed by the HRP substrate ECL solution (1 M Tris-HCl, pH 8.5, 250 mM Luminol, 90 mM Coumaric acid and 30% Hydrogen peroxide). In this study, we have investigated the expression of the key molecules involved in the mitochondrial and extra-mitochondrial apoptotic pathway [41].

### Effect of caspase inhibition on the pro-apoptotic activity of 1j

The effect of caspase inhibition on the pro-apoptotic activity of **1j** was evaluated by the MTT cell viability assay [42]. Prior to **1j** exposure, the cells were pre-treated with Z-VAD-FMK pan-caspase inhibitor (50 μM) for 1 hour. The cells were then treated with 20μM **1j** for 24 hours. In another set, cells were treated with **1j** only (without the pre-treatment of Z-VAD-FMK). Finally, cell viability was determined as described earlier.

### Flow cytometric analysis of cell cycle

Cell cycle progression of CCF-4 cells was determined flow cytometrically following the protocol of Riccardi et al. [43]. Briefly, the cells were synchronized and cultured in RPMI medium. Then the cells were exposed to **1j** and CDDP respectively, at desired concentrations. Next, the cells were harvested after 24 hours, washed with cold PBS and fixed for 4 hours in 70% ethanol (at 4°C). The cells were then subjected to centrifugation (1500 rpm, 4 minutes)followed by washing (with cold PBS containing 2% FBS) and treatment with ribonuclease. Finally the cells were incubated for 30 minutes with 50 μg/ml propidium iodide at 37°C. The fluorescence was measured using a flow cytometer and analyzed by the Cell Quest program.

### Wound healing assay

CCF-4 cells were seeded in a 6 well plate under proper conditions as mentioned above. After 24 hours of plating, a wound was gently made by scratching the surface with the help of a 200 μl pipette tip. Then half the wells were left untreated and half were treated with 20 μM of **1j**. The cells were photographed by a phase contrast microscope after 0, 8, 16 and 24 hours of **1j** exposure [44–46]. The data was quantified using the Image J software.

### Colony forming assay

Colony forming assay or the clonogenic assay was performed to evaluate the ability of the cancer cell to grow into a colony after the desired treatment with the **1j**. The assay was performed according to the protocol of Franken *et al* [47]. Briefly, 0.3 x 10^6^ cells were plated in a culture dish. After treatment, the CCF-4 cells were trypsinized and replated under normal culture condition. The culture dish was then incubated for 14 days under normal culture conditions. Finally the cells were washed with PBS and stained with a 0.5% crystal violet solution containg 6.0% glutaraldehyde. The culture dish was then photographed and quantified using the Image J software.

### Statistical analysis

After performing three independent experiments, results have been expressed as their mean data (±SD). The statistical evaluation has been done by the means of one-way analysis of variance (ANOVA), and Tukey test was used to compare the group means. A p-value less than 0.05 were considered as statistically significant.

## Results

### Bis-lawsone derivatives are cytotoxic to cancer cells

**Fig 2:**
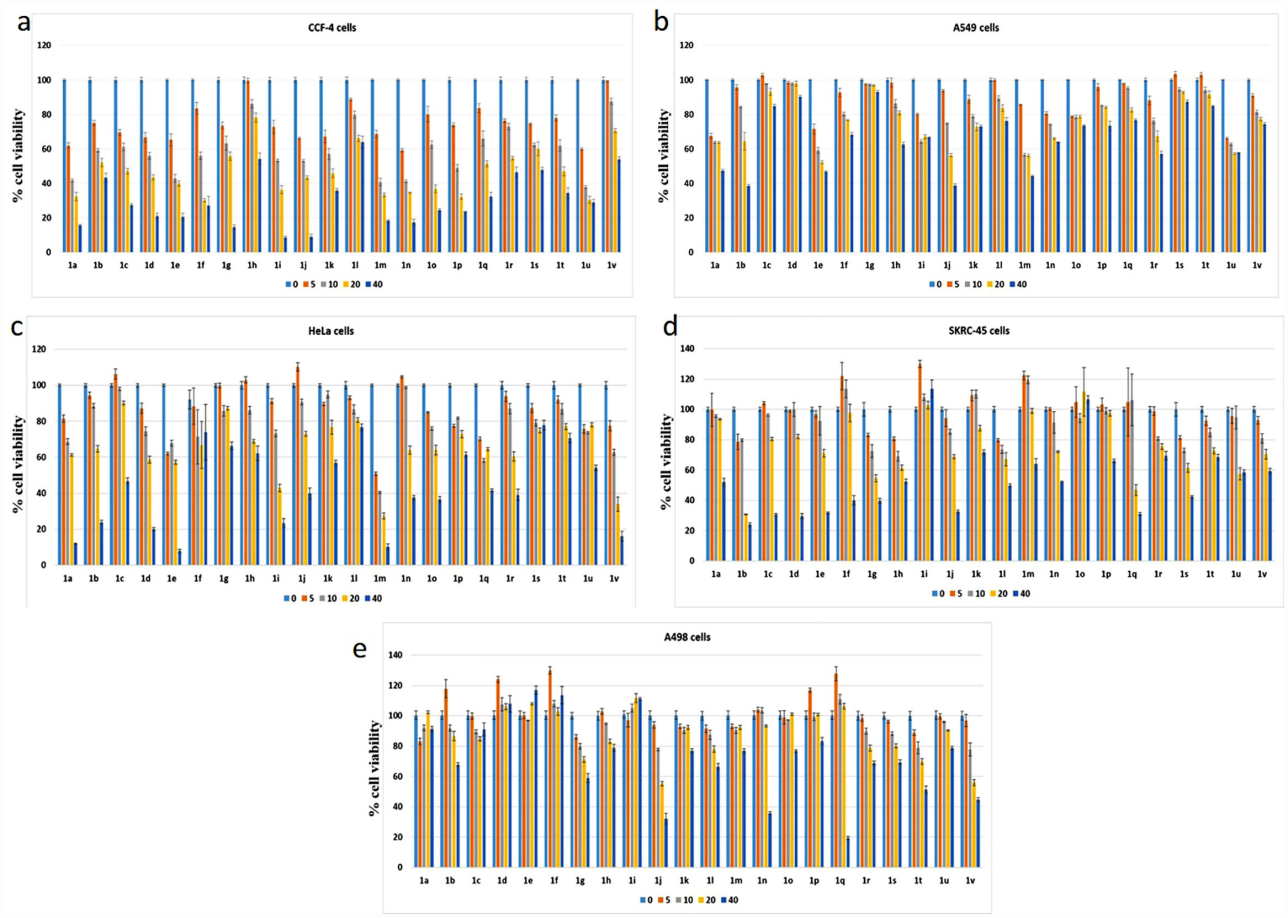
The dose dependent effect on cell viability of all the 22 bis-lawsone derivatives on the 5 human cancer cell lines. The derivatives were exposed to the different cells at a dose of 5, 10, 20 & 40 μM. a. CCF-4 cells, b. A549 cells, c. HeLa cells, d. SKRC-45 cells, e. A498 cells. Each point represents mean ±SD, n = 3 (number of plates).

To find out the optimum cytotoxic effect of these compounds (**1a**-**1v**), we have carried out the dose dependent MTT cell viability assay and the results have been presented in Fig 2. Out of 22 napthaquinone derivatives tested for their toxicity on different cell lines, six compounds **1b**, **1c**, **1d**, **1e**, **1j** and **1n** showed significant toxicity at less than 50 micromolar concentration in different cancer cells when compared to the normal NKE cells. Mostly CCF-4, HeLa and SK-RC-45 cell lines were sensitive to the compounds where as A498 and A549 cells remained less sensitive (Fig 2a–2e). The compounds **1b**, **1c**, **1d**, **1e**, **1j** and **1n** demonstrated remarkable anti-proliferative activity (low LC_50_ value) in CCF-4 and SK-RC-45 cells within a range of 7.29–17.96 μM and 16.98–43.14 μM respectively as calculated from the graph. Rest of the cell lines, exhibited LC50 at varying doses ranging from 30.49 to 83.54 μM. In renal carcinoma cell line, SK-RC-45, the anti-proliferative and/or cytotoxic effect of the compounds **1b**-**1g**, **1j**, **1n**, **1q**, **1s** and **1q** is very significant. At 20 μM dose, approximately 60% cell viability was observed and that was reduced to approximately 20–30% at 40 μM. Compounds **1i**, **1k**, **1o**, and **1p** on the other hand, did not affect the viability of these cells (Fig 2d). In case of A498, most of the compounds, however, did not show any significant effect except 1n, 1q, 1t and 1v which were found to be toxic only at the highest dose (40 μM), showed cell viability below 40% (Fig 2e). In CCF-4, **1b**, **1q** and **1s** shows around 50–60% cell viability at 40 μM dose. All other compounds in the series showed the LC_50_ at the dose of 10–20 μM (Fig 2a). Compound **1i and 1j** exhibited the most significant anti-proliferative effect in CCF-4 cells. In case of A549, most of the compounds had moderate cytotoxic effect as they exhibited approximately 20% inhibition in cell viability at the highest dose (40 μM). All the working concentrations of **1c**, **1d**, **1g 1q, 1s** and **1t** showed almost no effect on A549 (Fig 2b). Upon exposure of **1a**, **1b**, **1d**, **1e**, **1i, 1j, 1m, 1o, 1r** & **1v**, the HeLa cells showed gradual decrease in cell viability up to 40 μM concentration. Compounds **1f**, **1g**, **1p**, **1s**, **1t & 1u** did not show any drastic change in cell viability in the HeLa cells (Fig 2c).

### Effect of bis-lawsone derivatives on normal cells

**Fig 3:**
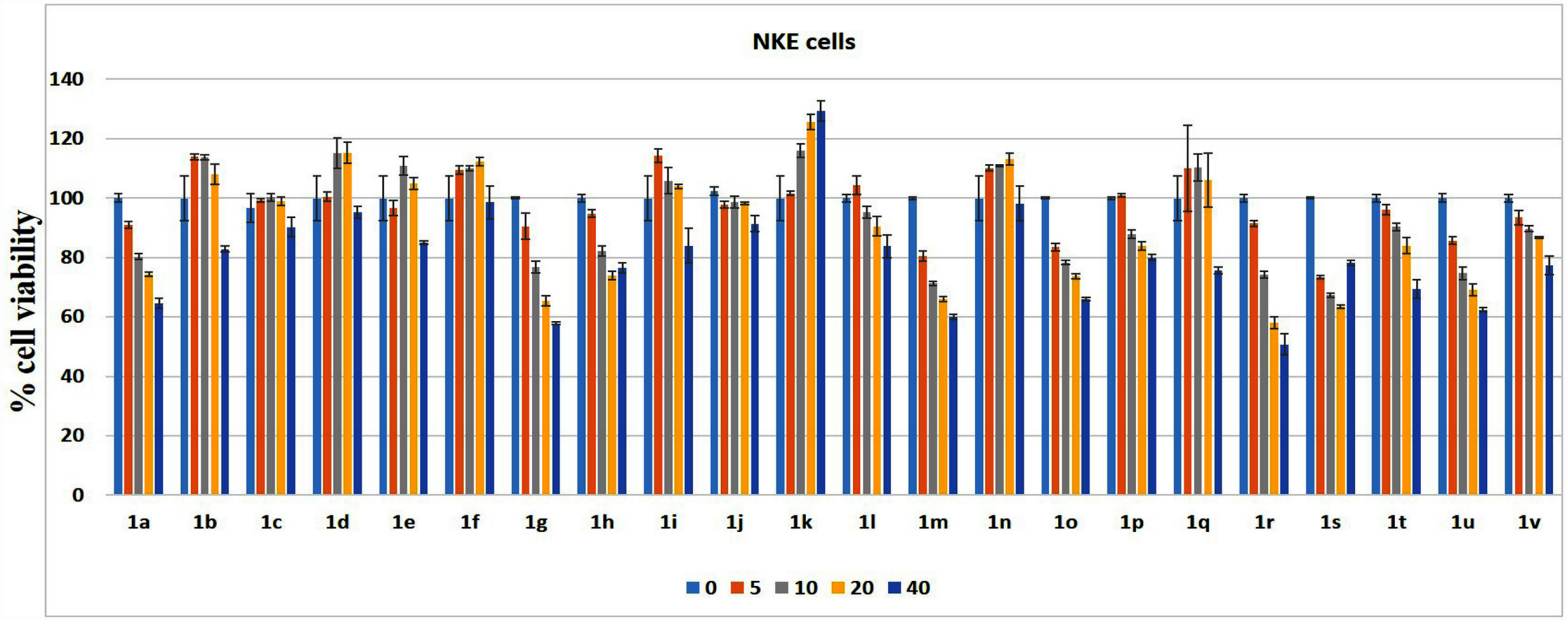
The dose dependent effect on cell viability of all the 22 bis-lawsone derivative on the human normal kidney epithelial cell line (NKE). The derivatives are exposed to the cell at a dose of 5, 10, 20 & 40 μM. Each point represents mean ±SD, n = 3 (number of plates).

Most of the compounds remained relatively non-toxic in NKE cells (the normal kidney cell line) showing LC_50_ values above 90.80 μM (to as high as in the range of millimolar; Fig 3), whereas compounds such as **1a**, **1g**, **1h**, **1i**, **1m, 1o, 1r-1u** exhibited toxicity to NKE cells with LC_50_ values as low as 51.31 μM.

### Differential cytotoxicity of 1j to the cancer cells

From the results of the rapid screening of the naphthoquinone derivative, **1j** derivative was found to be the most effective on the CCF-4 cells and it was not toxic to the normal cells. The calculated LC_50_ value of **1j** derivative on the CCF-4 cells is 17.18 μM, whereas in case of NKE cells the LC_50_ value is 222.12 μM. LC_50_ values of **1j** derivative on different cancer cells and NKE cells are comprehensively presented in Table 1.

**Table 1:** IC_50_ of the synthesized 1j derivative on six different cell lines.

*Cell lines*	CCF-4	A549	HeLa	SKRC-45	A498	NKE
IC_50_ value of 1j (μM)	**17.18 ± 0.53**	29.57 ± 1.77	34.32 ± 1.80	30.31 ± 3.66	27.47 ± 12.33	**222.12 ± 6.30**

**Fig 4:**
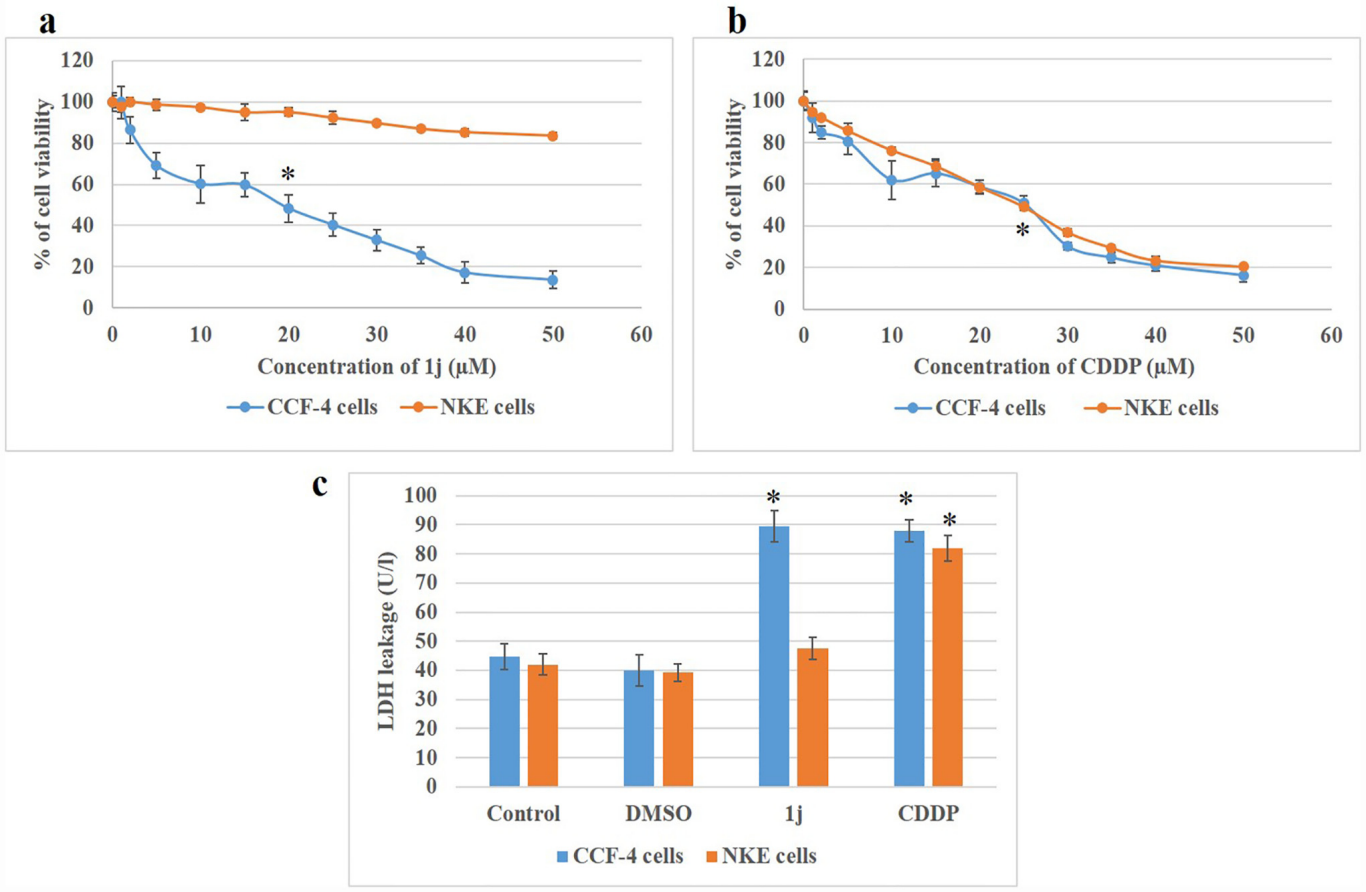
The dose dependent cytotoxic effect of 1j bis-lawsone derivative and CDDP on CCF-4 and NKE cells. a. Effects of different concentrations of **1j** ranging from 0 μM to 50μM, on both the cells viability by MTT assay. b. Effects of different concentrations of CDDP ranging from 0 μM to 50μM, on both the cells viability by MTT assay. c. Cytotoxic effect of both **1j** and CDDP on both the cancerous and normal cell line by LDH leakage assay. Each point represents mean ±SD, n = 3 (number of plates). ‘‘*” represents the significant difference compared to the control cells. (P* < 0.05).

Furthermore, the cytotoxicity of **1j** derivative was compared with CDDP. It was found that **1j** derivative is selectively toxic to the glioma cells compared to NKE cells (Fig 4a). When CCF-4 and NKE cells were treated with CDDP, the LC_50_ dose of CDDP was found to be 25 μM for both the cells (Fig 4b).

This result was further confirmed by assaying the level of LDH leakage from the cells. It was observed that the exposure of **1j** caused a leakage 89.5 U/l LDH from the CCF-4 cells. On exposure to CDDP, CCF-4 cells also exhibited significantly higher amount of LDH leakage (Fig 4c). In case of NKE cells, **1j** does not cause any cytotoxicity but exposure to CDDP causes a leakage of 81.8 U/l LDH.

### 1j differentially induces apoptosis in cancer cells and normal cells

**Fig 5:**
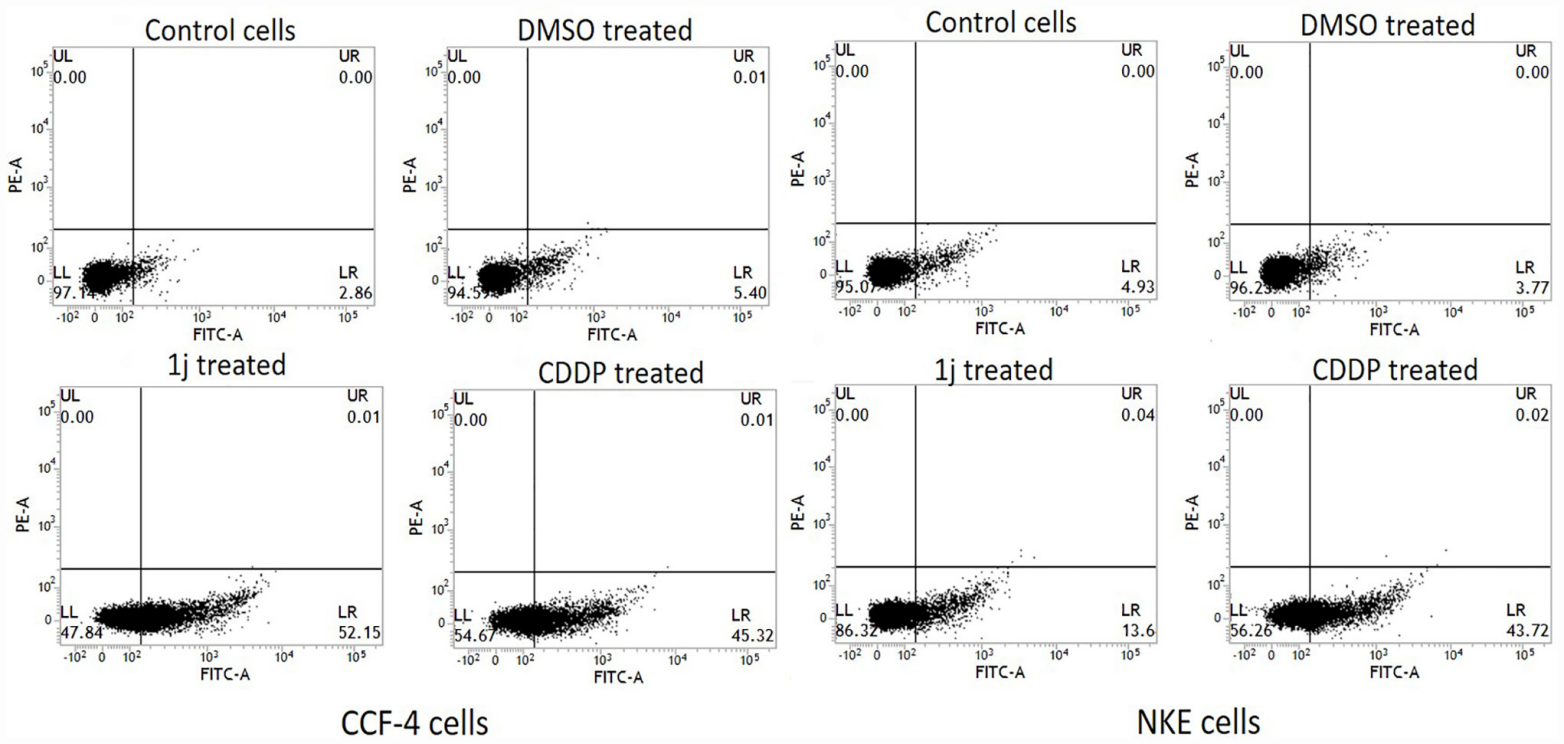
Detection of 1j and CDDP induced apoptosis on both the CCF-4 and NKE cells via Annexin V-FITC staining. The cells were incubated separately with desired molecules (20 μM **1j** and 25 μM CDDP) followed by staining with Annexin V-FITC and then the cells were flow cytometrically analyzed. Dual parameter dot plot of FITC-labelled Annexin V fluorescence (x-axis) has been shown in logarithmic fluorescence intensity. Data are representative of three independent experiments.

Annexin-V mediated FACS assay was done to confirm the mode of cell death induced by **1j**. The increase in the number of annexin-V tagged CCF-4 cells in **1j** and CDDP-exposed groups indicated that both the compounds induced apoptosis in these cells. In line with the previous observations, when NKE cells were treated with both of these molecules, unlike CDDP treated cells, **1j** exposed cells exhibited no such significant indication of apoptosis (Fig 5).

### 1j specifically induces intracellular ROS in cancer cells

**Fig 6:**
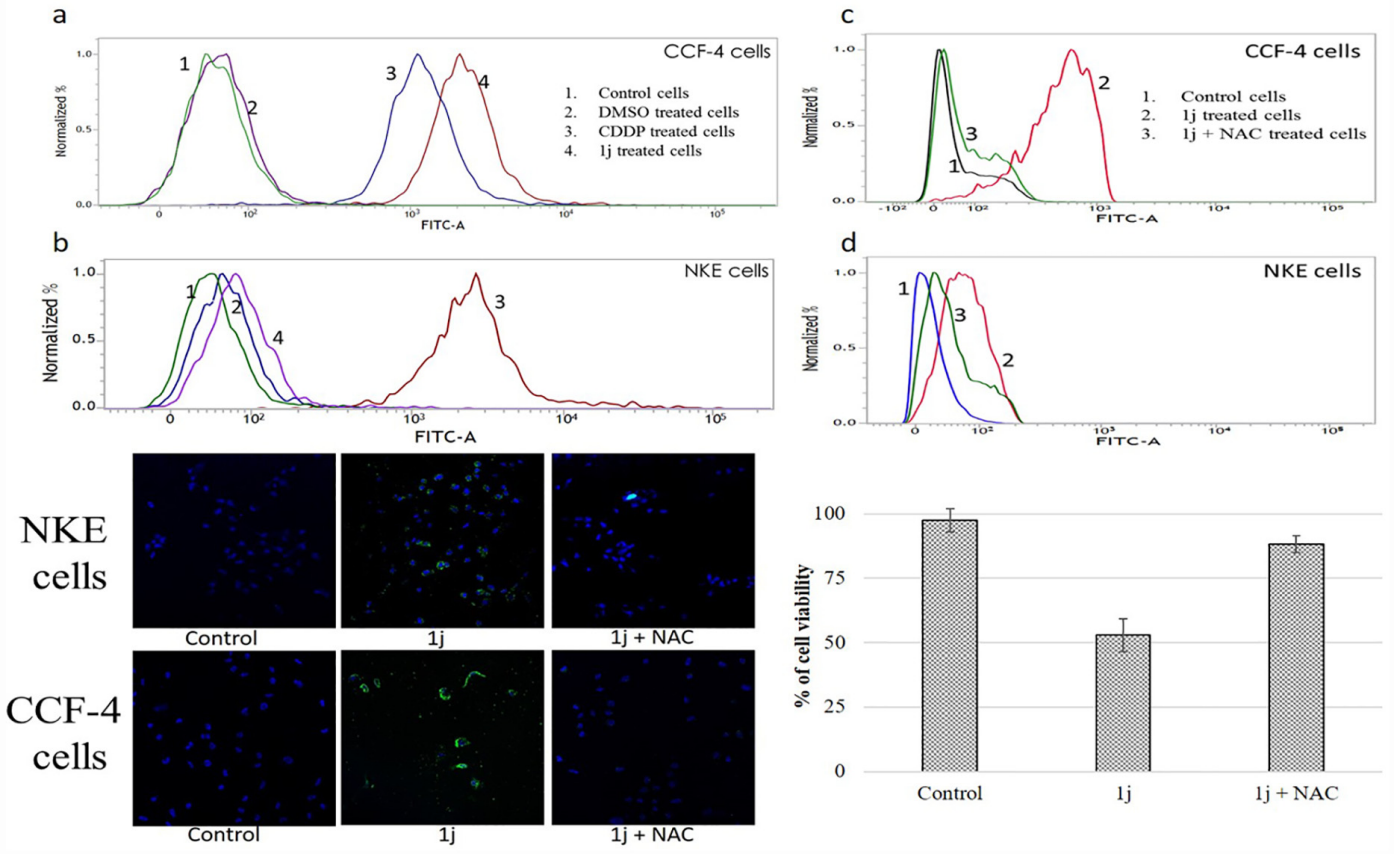
Effect of 20 μM 1j and 25 μM CDDP on ROS production in the cancerous and normal cells. a-b. DCFH-DA staining shows increased ROS production upon exposure of both the compounds in CCF-4 and NKE cells. c-d. Differential induction of ROS on CCF-4 and NKE cells upon exposure to 20 μM **1j** and 5mM NAC. e. Intracellular ROS production was detected by changes in the fluorescence intensity of DCF by fluoroscent microscopy (20X). Data are representative of three independent experiments. f. Effect on cell viability of CCF-4 cells upon exposure to 20 μM **1j** and 5mM NAC. Each column represents mean ±SD, n = 6. ‘‘*” represents the significant difference between the normal control and **1j** treated cells (P* < 0.05).

The level of intracellular ROS was measured in the control, **1j** and CDDP-treated both CCF-4 and NKE cells using flow-cytometric analysis. It was observed that the intracellular ROS level in the **1j-**exposed CCF-4 cells increased enormously compared to the control group and **1j-**exposed NKE cells. Elevation in the level of intracellular ROS was also observed in the case of CDDP-treated glioma cells and normal kidney cells (Fig 6a and 6b). When the experimental cells were pre-treated with 5 mM NAC, a potent antioxidant that scavenges ROS, the elevation in the ROS level was inhibited in the **1j** treated CCF-4 cells (Fig 6c and 6d). This data was further confirmed by the fluorescent micrographs. These micrographs also indicate that **1j** selectively enhanced the level of intracellular ROS in glioma cells compared to the normal cells. We found that the intensity of green fluorescence (produced due to ROS) was significantly less in NKE cells compared to CCF-4 cells treated with **1j**. For both the cell types, NAC pre-treatment inhibited the elevation in intracellular ROS level (Fig 6e). Finally to confirm the role of oxidative stress in **1j** mediated CCF-4 cell death, cell viability assay was performed. We found that the NAC pre-treatment significantly inhibited the cytotoxic effect of **1j** in CCF-4 cells (Fig 6f).

### 1j exposure alters the activity of antioxidant enzymes in CCF-4 cells

**Fig 7:**
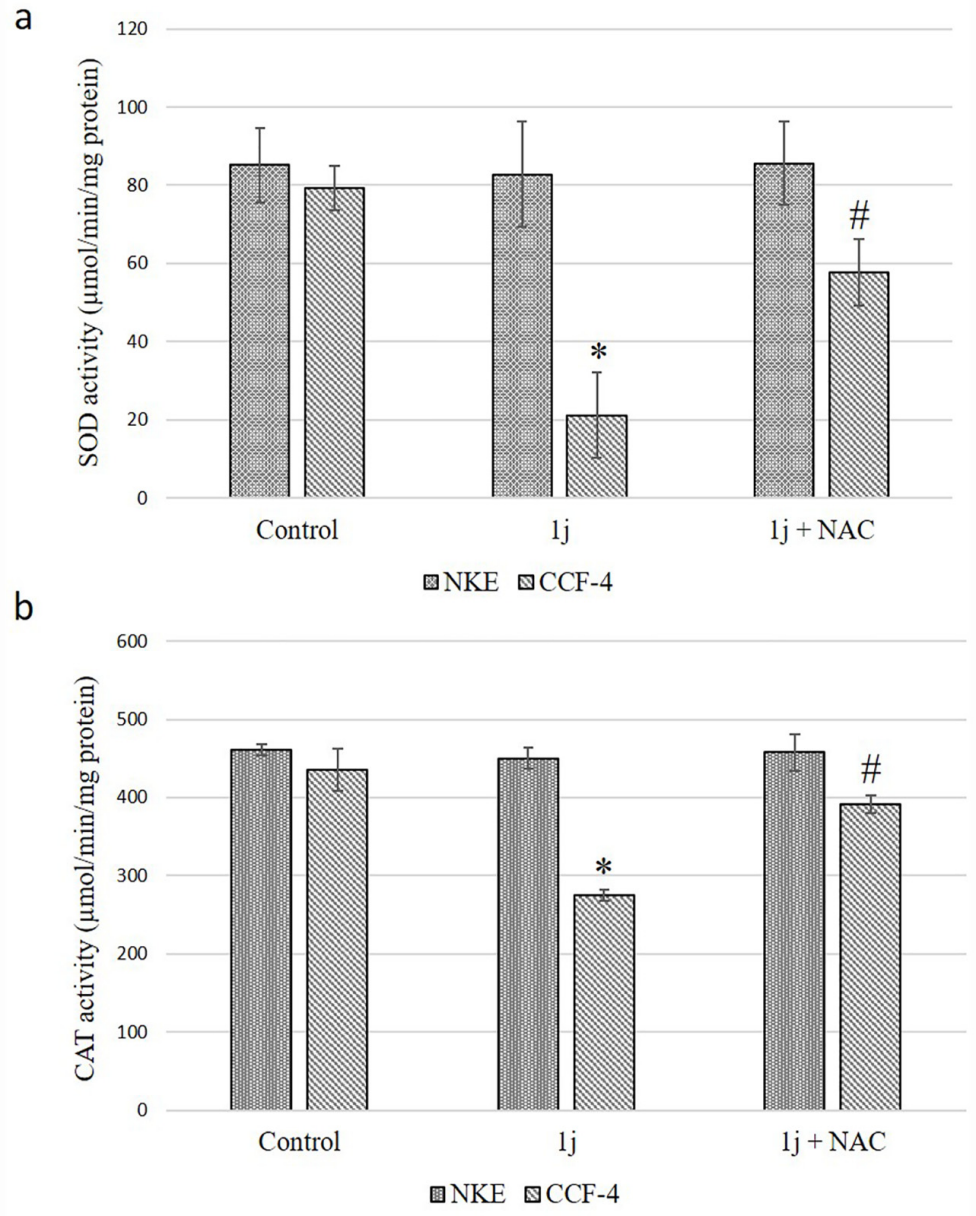
a. Effect of 20 μM **1j** derivative on SOD activity activities in CCF-4 and NKE cells. b. Effect of **1j** derivative on CAT activity activities in CCF-4 and NKE cells. Each column represents mean ±SD, n = 6. ‘‘*” represents the significant difference between the normal control and **1j** treated cells and ‘‘#” represents the significant difference compared to the CDDP treated cells. (P* < 0.05).

**1j** and CDDP exposure caused a significant reduction in the activity of antioxidant enzymes superoxide dismutase (SOD) and catalase (CAT) in CCF-4 cells. In NKE cells, the activities of these enzymes were not significantly affected upon **1j** exposure. In CCF-4 cells 5mM NAC pre-treatment inhibited the cytotoxic effect of **1j** and maintained the activities of these enzymes compared to the untreated cells (Fig 7).

### 1j exposure alters the oxidative stress markers in CCF-4 cells

**Fig 8:**
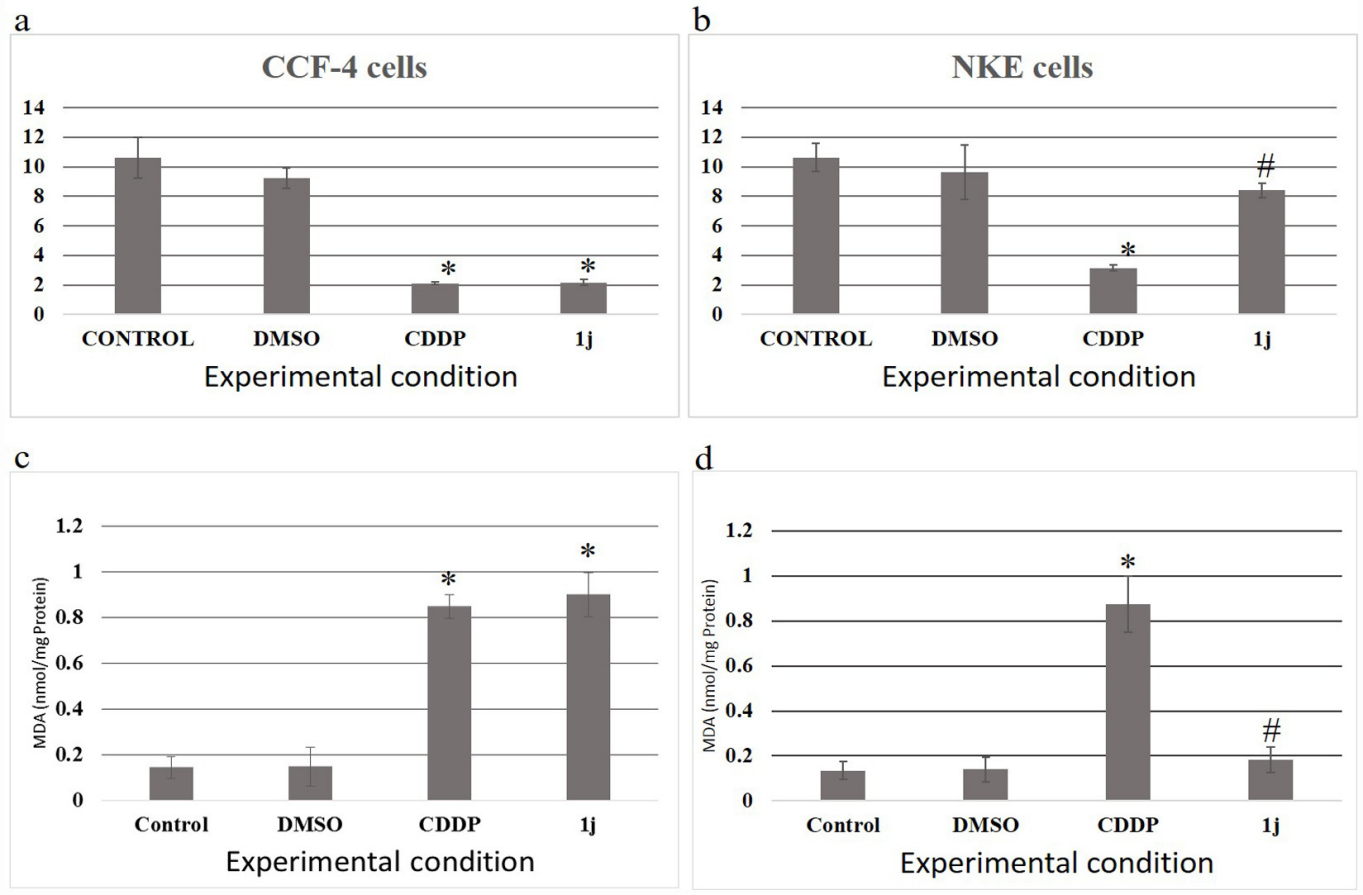
a. Effect of 20 μM **1j** and 25 μM CDDP on Glutathione (GSH), oxidized Glutathione (GSSG), and their ratio in CCF-4 cells. b. Effect of **1j** and CDDP on Glutathione (GSH), oxidized Glutathione (GSSG), and their ratio in NKE cells. c-d. Effect of **1j** and CDDP on lipid peroxidation in both CCF-4 and NKE cells. Each column represents mean ±SD, n = 6. ‘‘*” represents the significant difference between the normal control and **1j** treated cells and ‘‘#” represents the significant difference compared to the CDDP treated cells. (P* < 0.05).

**1j** and CDDP exposure caused a significant reduction in the level of reduced glutathione (GSH) along with an increased level of oxidized glutathione (GSSG) in CCF-4 cells. NKE cells upon being exposed to **1j** did not result in any oxidative stress conditions, whereas CDDP exposure resulted reduced GSH-GSSG ratio in NKE cells (Fig 8a and 8b). In line with this data, it was observed that **1j** exposure significantly increased the level of malondialdehyde (MDA) in the glioma cells barring the normal cells (Fig 8c and 8d), which clearly indicates that **1j** could selectively induce oxidative stress in the glioma cells.

### 1j induces a disruption of mitochondrial membrane potential

**Fig 9:**
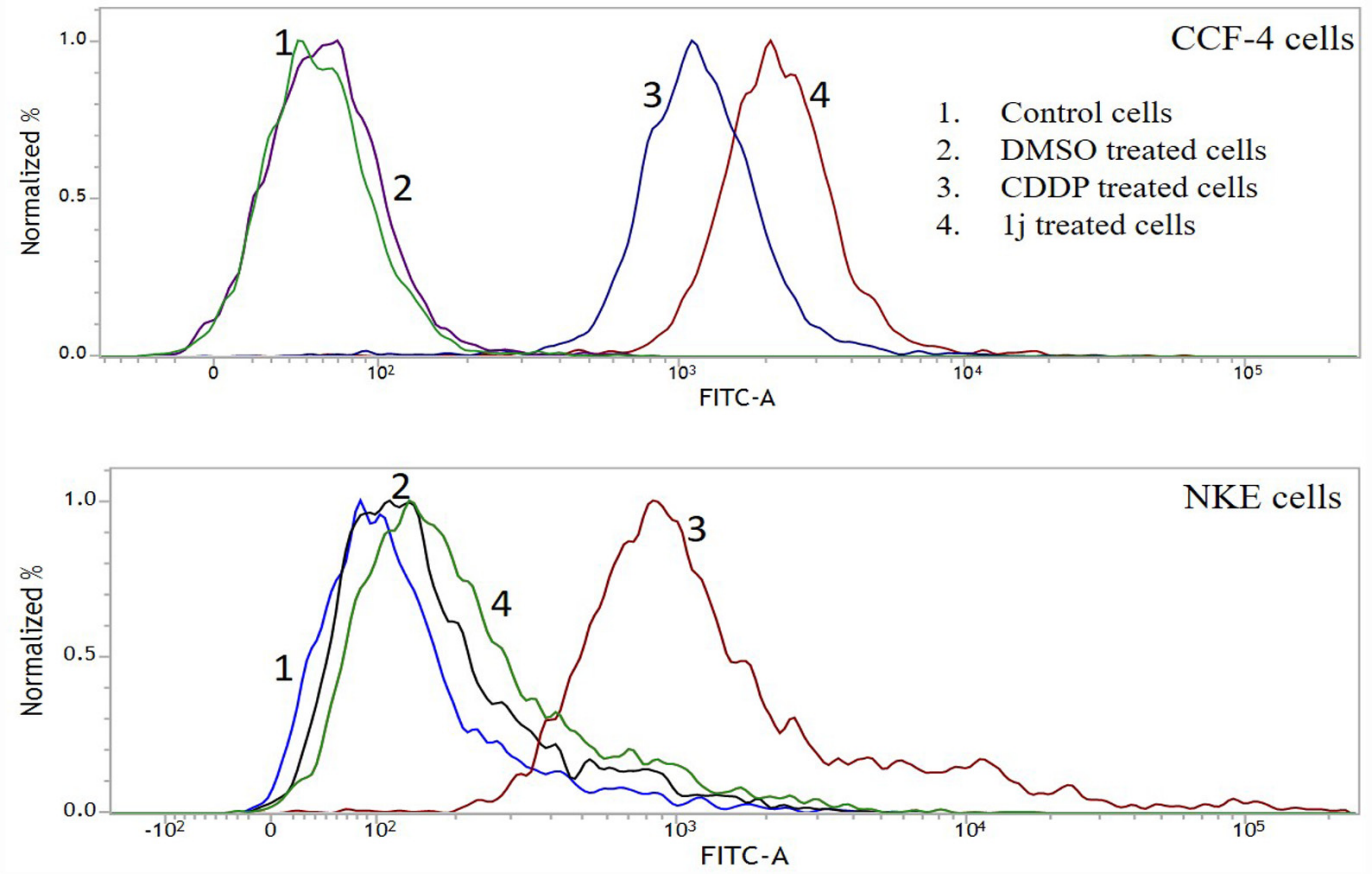
Effect of 20 μM 1j and 25 μM CDDP on mitochondrial membrane potential in CCF-4 cells and NKE cells as shown by the monomeric green fluorescence of the JC-1 dye. Data are representative of three independent experiments.

After confirming apoptosis as a mode of cell death in the **1j-**exposed CCF-4 cells, the mitochondrial membrane potential (MMP) was determined with the help of FACS using JC-1 dye. An increase in green fluorescence with a concurrent decrease in the red fluorescence was observed in the **1j** and CDDP exposed CCF-4 cells. This observation indicated a significant decrease of MMP in the glioma cells. Unlike cisplatin exposed NKE cells, **1j** did not cause any significant change in the oxidation–reduction potential of mitochondria in NKE cells. From this data, we could infer that **1j** derivative was likely to induce apoptotic cell death via mitochondrial dysfunction (Fig 9).

### 1j differentially modulates the expression of the regulatory proteins of intrinsic and extrinsic pathway of apoptosis

When a cell undergoes apoptosis, intracellular Bax/Bcl2 ratio increases, as the expression of Bax increases and Bcl2 decreases. It was observed that **1j** and cisplatin-exposed CCF-4 cells had potentially increased Bax/Bcl2 ratio than the untreated cells. Unlike CDDP, the Bax/ Bcl2 ratio was positively maintained in **1j** exposed NKE cells. It was previously observed that **1j** caused an alteration in the mitochondrial membrane potential (MMP) differentially in CCF-4 and NKE cells, and in line of this data we found that **1j** exposure elevated the level of cytosolic cytochrome C, but **1j** exposure did not make any significant change for the same in NKE cells.

**Fig 10:**
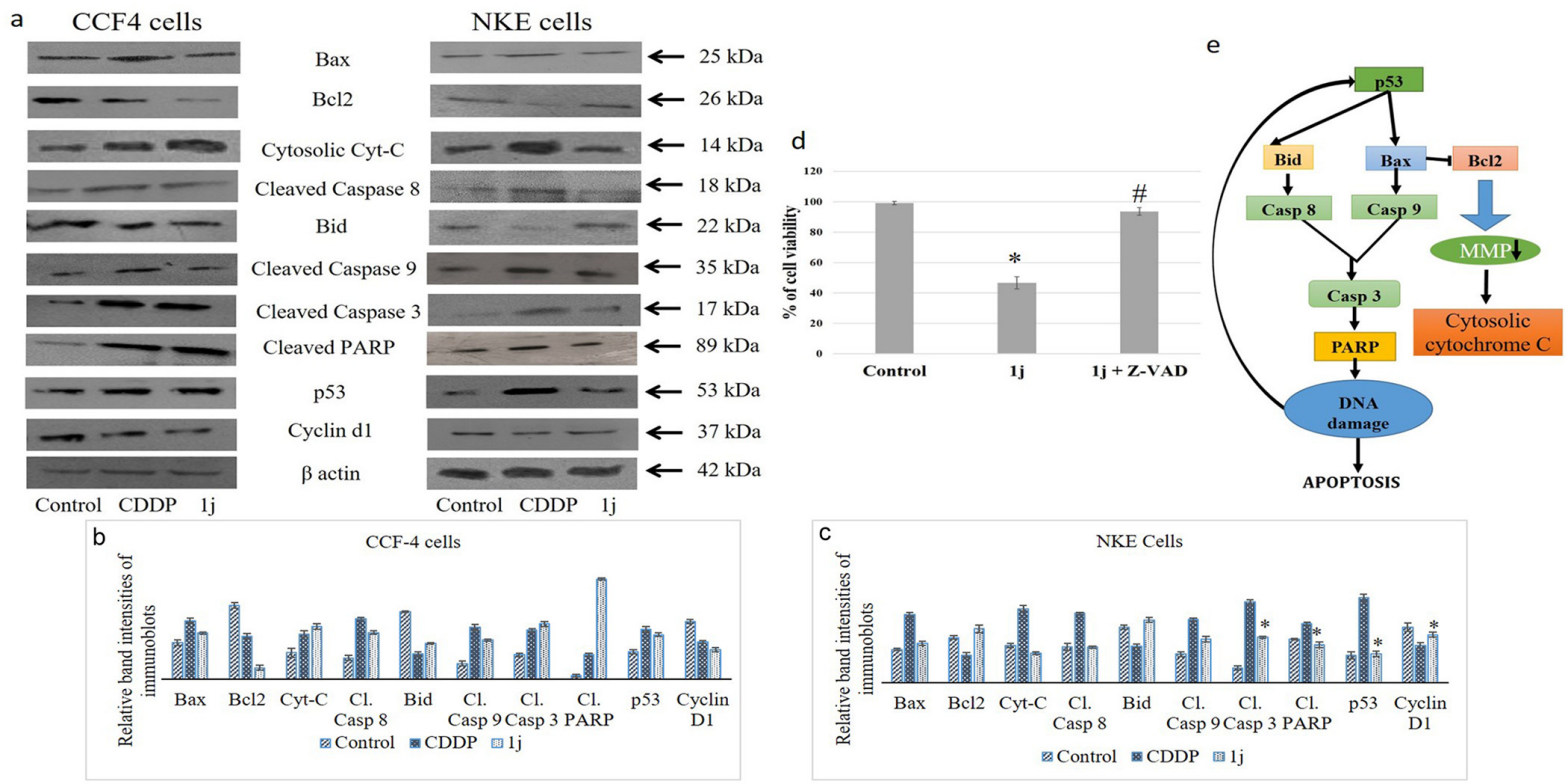
a. Immunoblot analysis of different signalling molecules considered in the study. β actin was used as an internal control. Data are representative of three independent experiments. b-c. Densitometric analysis of the respective immunoblots. d. Effect on cell viability upon **1j** exposure in the presence or absence of the pan-caspase inhibitor z-VAD-fmk. All data are mean ± SD, for 3 independent experiments and were analyzed by one-way ANOVA. ‘‘*” represents the significant difference between the normal control and **1j** exposed cells. e. The schematic representation of the regulation of various signaling molecules due to **1j** and CDDP exposure in CCF-4 and NKE cells.

Moreover, on exploring the molecules of the extrinsic pathway we observed that **1j-**exposure induced the expression of caspase 8, and thus a decreased level of Bid was observed in its downstream. We also found an increased expression level of different proapoptotic caspases like caspase 9 followed by caspase 3. Downstream of caspase 3, we observed an increased expression of cleaved PARP, which indicated the DNA damage and eventually cell death (Fig 10a).

The pro apototic activity of the novel derivative was found to be dependent upon caspase activation (Fig 10d). Exposure to **1j** derivative reduced the cell viability by 53.4% than the control cells, but when the CCF-4 cells were subjected to the same treatment in presence of the pan caspase inhibitor (z-vad) the effect was almost reversed and cell viability was found to be 93.7%. These results suggested that exposure of **1j** induced a caspase dependent apototic pathway in the glioma cells.

### 1j differentially activates p53 and cell cycle regulatory proteins in cancerous and normal cell lines

Development of cancer is a multistep process that involves an activation and inactivation of different oncogenes and tumor suppressor genes respectively. Hence, we checked the expression of p53, a tumor suppressor protein and Cyclin D1 that regulates the cell cycle. In this study, we observed that **1j** induced the expression of p53 in the CCF-4 cells. The induced expression of p53 in **1j** treated cells was found similar to cisplatin exposed cells, However, unlike cisplatin, **1j** molecule did not induce the expression of p53 in NKE cells.

Cyclin D1 is responsible for the progression of the cell cycle at the G0/G1 stage. It was observed that cisplatin and **1j** exposure down-regulated the expression of Cyclin D1 in CCF-4 cells. However, exposure to **1j** derivative in NKE cells did not cause any change in cyclin D1 expression (Fig 10a).

### 1j potentially arrests the cell cycle progression

**Fig 11:**
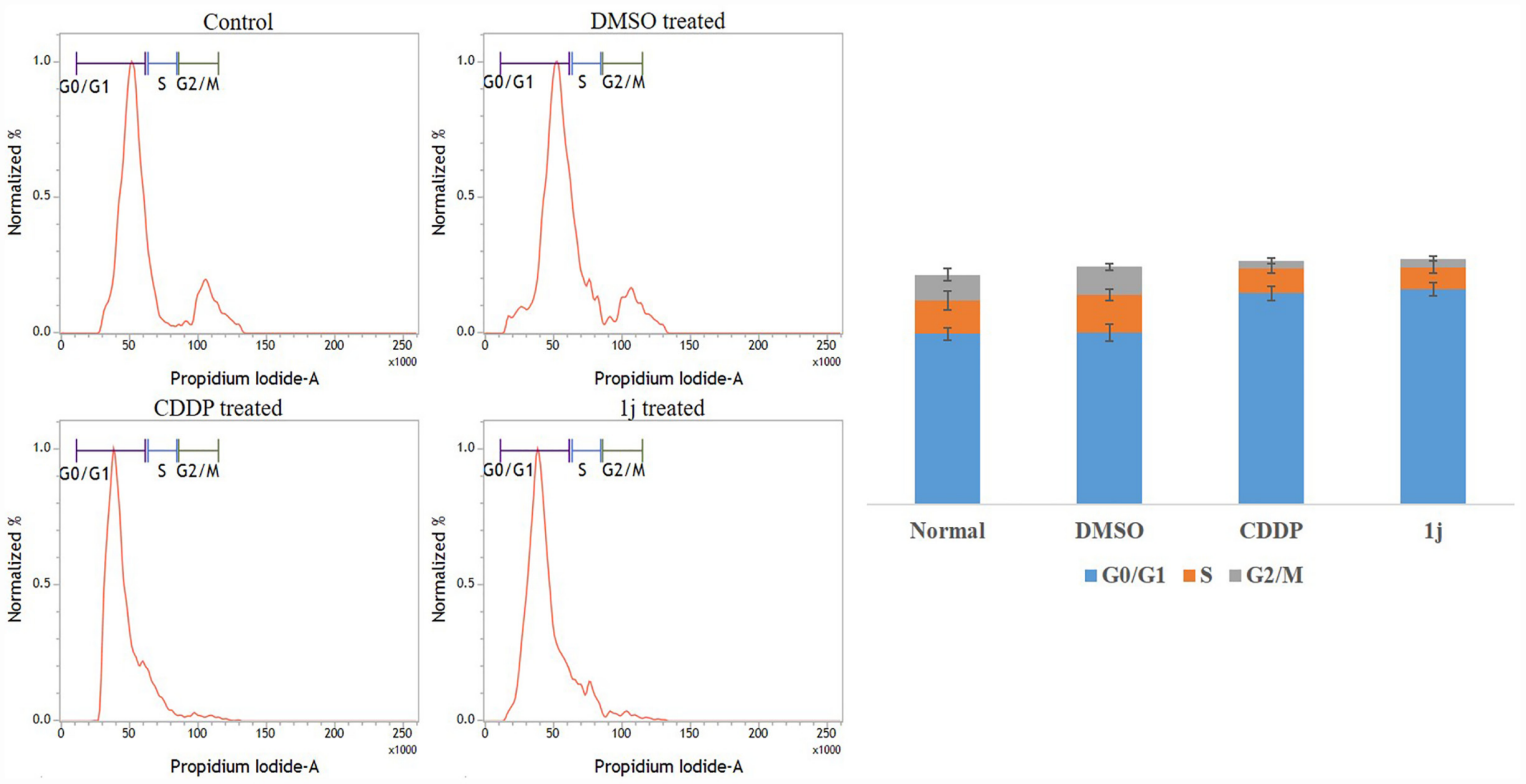
Effect of 20 μM 1j and 25 μM CDDP on cell cycle progression in CCF-4 cells. It was observed that **1j** derivative and CDDP induces cell cycle arrest at G0-G1 phase. Data are representative of three independent experiments. All data are mean ± SD, for 3 independent experiments and were analyzed by one-way ANOVA. ‘‘*” represents the significant difference between the normal control and **1j** exposed cells.

Flow cytometric analysis demonstrated inhibitory effects of **1j** and cisplatin on cell cycle progression in CCF-4 cells. CCF-4 cells were treated with desired concentration of **1j** and cisplatin, as mentioned earlier. It was observed that both the compounds significantly induced cell cycle arrest in G0-G1 phase in the glioma cells (Fig 11).

### 1j inhibits the cellular motility of CCF-4 cells

**Fig 12:**
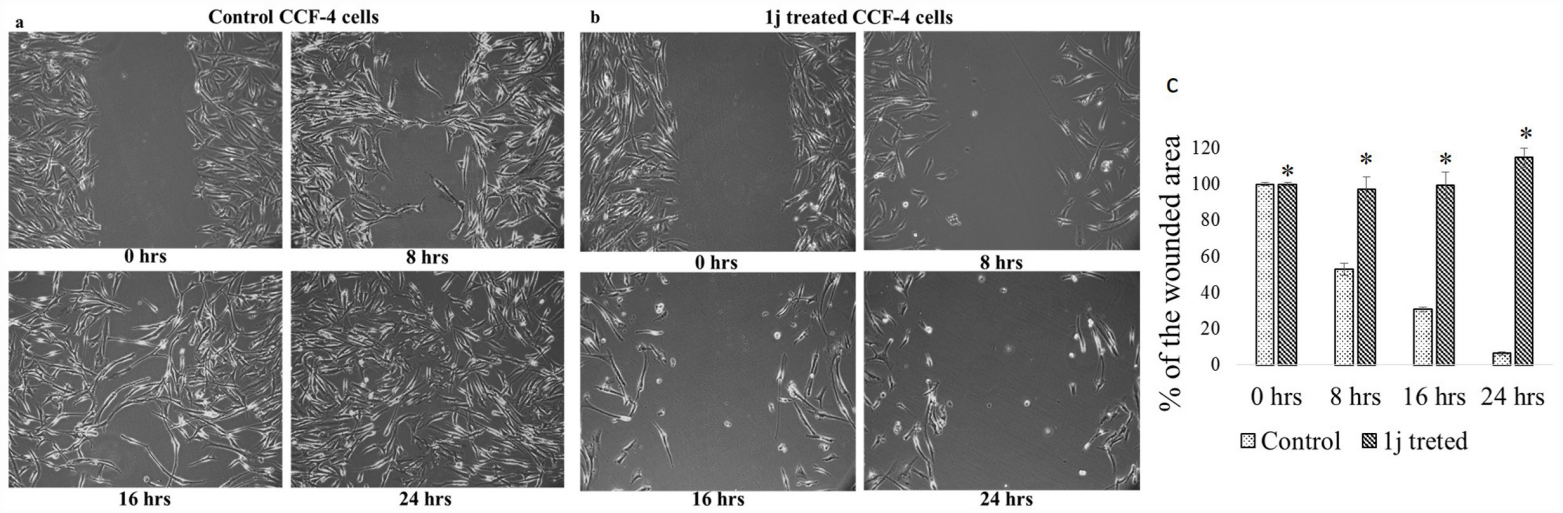
Effect of 20 μM 1j derivative on cell migration was observed by a phase contrast microscope (100X). a. untreated CCF-4 cells b. 1j exposed CCF-4 cells. Data are representative of three independent experiments. All data are mean ± SD, for 3 independent experiments and were analyzed by one-way ANOVA. ‘‘*” represents the significant difference between the normal control and **1j** exposed cells.

**Fig 13:**
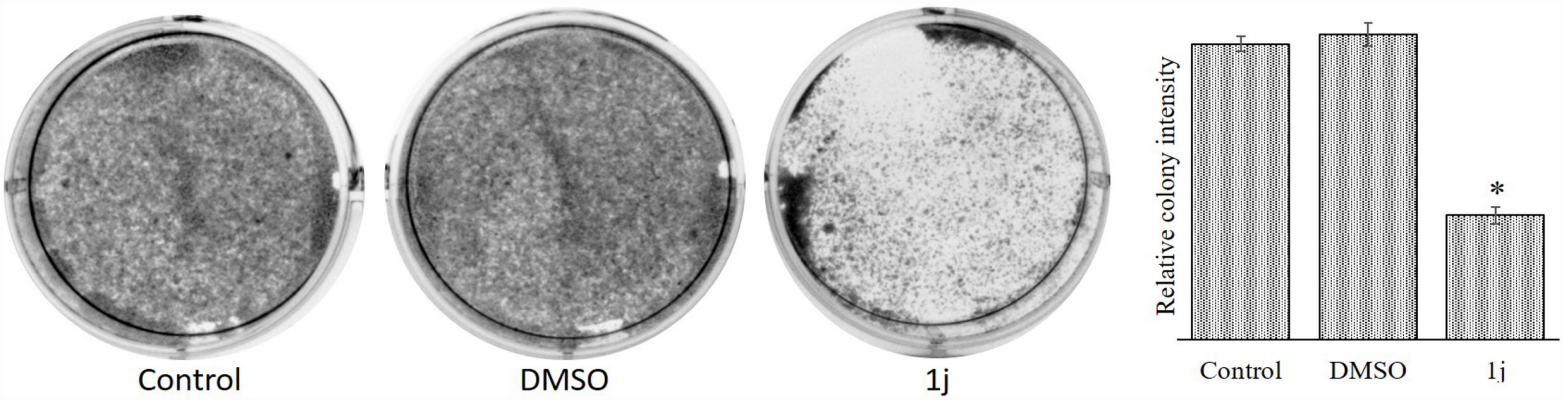
Effect of 20 μM 1j derivative on colony forming ability of CCF-4 cells post treatment. Data are representative of three independent experiments. All data are mean ± SD, for 3 independent experiments and were analyzed by one-way ANOVA. ‘‘*” represents the significant difference between the normal control and **1j** exposed cells.

To investigate whether proliferative changes in **1j** treated cells affected the cellular motility of CCF-4 cells, wound healing assay and colony forming assay were performed. It was observed that the untreated cells gradually grew up and filled the wounded region (Fig 12), whereas cells exposed to 20 μM **1j** derivative did not grow much in the wounded region and the cell density was significantly decreased (Fig 12). Thus, it can be inferred from this observation that the wound healing ability or the movement of the glioma cells was decreased after **1j** exposure. As expected, exposure to **1j** significantly reduced the colony formation in the CCF-4 cells compared with the cells in control group (Fig 13). Compared to the well-developed colonies observed in control cells, **1j** treated cells formed fewer colonies and were relatively smaller in size. Therefore, these results suggest that exposure to **1j** is critical for the oncogenic property of glioma cells.

### 1j alters the PI3K/Akt/mTOR protein expressions in CCF-4 cells

**Fig 14:**
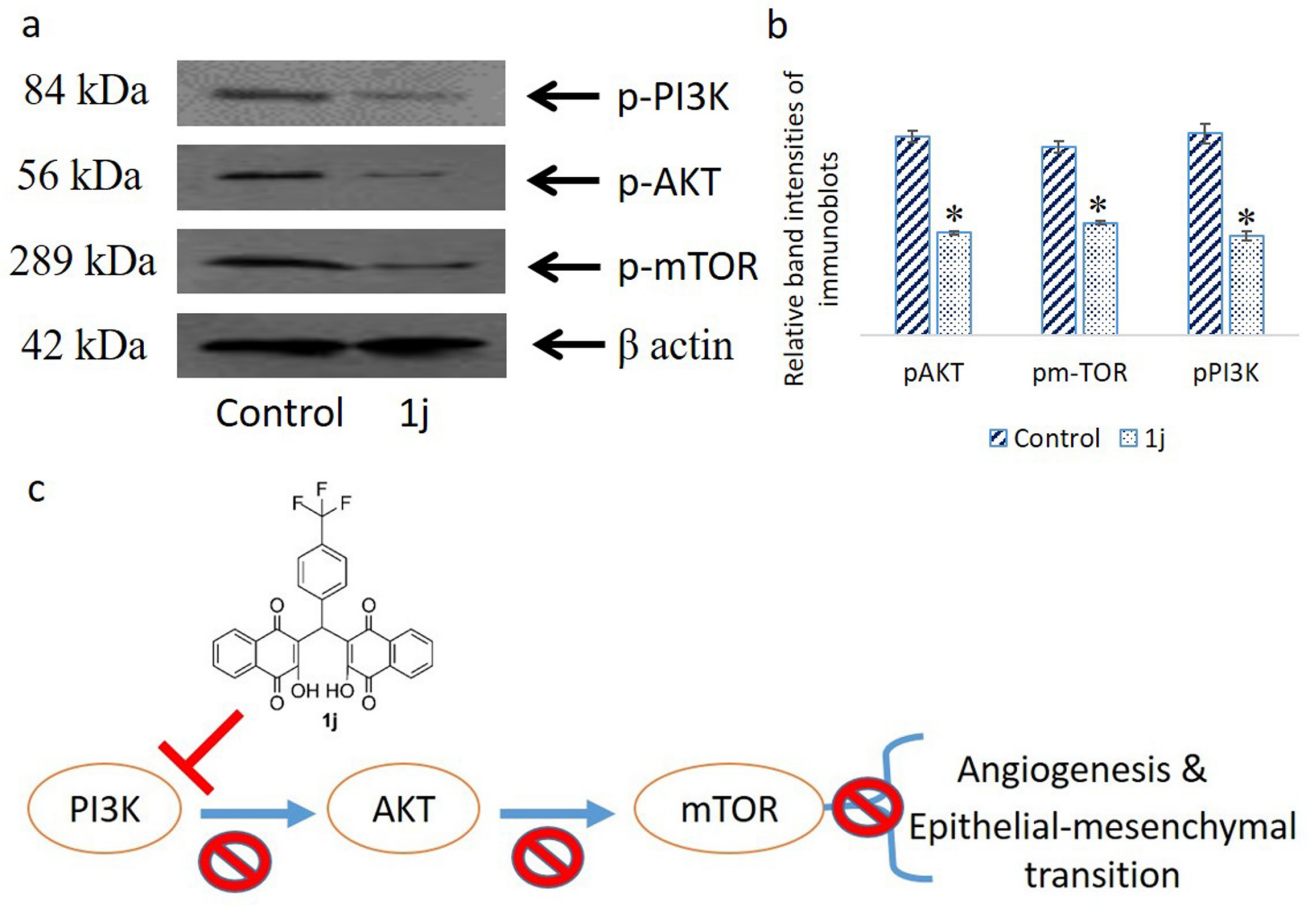
a. Immunoblot analysis of different signalling molecules considered in the study. β actin was used as an internal control. Data are representative of three independent experiments. Control: untreated cells, 1j: 20 μM **1j** exposed cells. b. Densitometric analysis of the respective immunoblots. All data are mean ± SD, for 3 independent experiments and were analyzed by one-way ANOVA. ‘‘*” represents the significant difference between the normal control and **1j** exposed cells. c. Schematic representation of the most probable signalling cascade moduated due to **1j** exposure.

PI3K/AKT/mTOR signalling pathway proteins play significant role in cell proliferation and metabolism and in cancer associated pathophysiological conditions. These proteins facilitate angiogenesis, cellular invasion and metastasis. In order to evaluate the effect of **1j** on the expression of these cell survival proteins in CCF-4 cells, immunoblotting experiments was performed. It was observed that **1j** significantly downregulated the expression of these proteins compared to the control cells (Fig 14).

## Discussion

In this study, **1j-**exposure significantly inhibited the proliferation of human glioblastoma cells in a dose-dependent manner and exhibited relatively much lower toxicity in normal cells compared to cisplatin. The bis-lawsone derivative, **1j**, dose-dependently induces cytotoxicity in all types of cancer cells used in this study, and is found to be most effective on the glioma cells. Apart from the MTT cytotoxicity assay, we have confirmed the cytotoxic nature of **1j** derivative by performing LDH cytotoxicity assay. The cytotoxic nature of this bis-lawsone derivative has also been compared to another potent anti-cancer drug, cisplatin [48,49]. By performing both the cytotoxicity assays, we found that **1j** caused 50% cell death at 20 μM dose while the same level of cytotoxicity is induced by cisplatin at 25 μM. Interestingly, we found that the screened bis-lawsone derivative is selectively toxic to the cancer cells but cisplatin is toxic to both the cancer and normal cells. For NKE cells, the LC_50_ dose was calculated as 90.80±12.33 μM (Fig 3). After confirming the cytotoxic nature of **1j** derivative, we wanted to determine the mode of cell death it induced in the CCF4 cells. It is a well-established fact that during the early onset of apoptosis, cells translocate the phosphatidylserine (PS) moieties present on the inner surface of the plasma membrane to its exterior. Once on the outer cell surface, PS moieties can be detected flow cytometrically by staining with a fluorescent conjugate of a protein (Annexin V) with high affinity for PS [50]. Detection of Annexin V by FACS analysis indicated that **1j** specifically inhibited cell proliferation in CCF-4 cells by the induction of apoptosis. In this experiment, we again found that the bis-lawsone derivative can effectively differentiate between the normal and cancer cells although cisplatin fails to do the same. While exploring the molecular mechanism behind the cytotoxicity of **1j** derivative, intracellular ROS level was measured to determine if oxidative stress is playing a role in inducing apoptosis in CCF-4 cells [51,52]. It was observed that like cisplatin, **1j** derivative also induced the elevation of ROS in the glioma cells, but it did not make any significant change in the ROS level in the normal cells. The oxidative stress mediated cytotoxic effect of **1j** derivative was further confirmed by pre-exposure to NAC, a well-known antioxidant molecule. Intracellular ROS level and cell viability were measured to investigate the role of oxidative stress in **1j** induced cell death in the glioma cells. It was found that NAC pre-treatment does not have any effect on the NKE cells but it reduces the cytotoxic effect of **1j** derivative in CCF-4 cells. From these results we can infer that 1j induced cell death in CCF-4 is mediated by oxidative stress. The same inference is reflected again when we determined the redox status of the cells by quantifying the GSH-GSSG and MDA level in both the cell types [53–55]. Next, to observe the mitochondrial health and the role of mitochondria in inducing apoptosis [56,57], we determined the mitochondrial membrane potential by using JC-1 stain employing a flow cytometer. By quantifying the intensity of green and red fluorescence, we found that **1j** specifically decreased the MMP in glioma cells but not in NKE cells. From this data, it was also indicated that mitochondria might play a crucial role in the progression of apoptotic pathway induced by the bis-lawsone derivative [58].

Literature suggests that most of the antitumor therapies induce apoptosis of cancer cells [59]. Between the two pathways of apoptosis, the intrinsic pathway is mediated by mitochondria and the extrinsic pathway is mediated by the death receptors [60]. Mitochondria mediated apoptotic signaling pathway can be activated via the modulation of several antiapoptotic and proapoptotic proteins of the Bcl-2 family [60] and is regulated by maintaining the balance between the expression of antiapoptotic Bcl-2 and proapoptotic Bax proteins. A decreased Bcl-2/Bax ratio indicates an enhanced pro-apoptotic effect [61]. In our study, following the **1j** exposure, we found a decreased Bcl-2 and an increased Bax expressions. Moreover, we found that upon exposure of **1j** to the CCF-4 cells, caspase-8 was activated and the expression of Bid was down-regulated. The decreased expression of Bid indicates the formation of active t-Bid [62]. Downstream of it, other caspases like caspase 9 and caspase 3 are activated [63]. Finally, we also observed an increased level of cleaved PARP in the **1j** exposed CCF-4 cells. Combining all these results, we conclude that this bis-lawsone derivative **1j** is inducing both the mitochondrial and extramitochondrial pathways of apoptosis in glioma cells. While observing the same molecular pathway on NKE cells, we found that the chosen derivative could not effectively induce any sort the apoptosis in them.

Cancer is a complex physiological disorder which disrupts the balance betwwen the activation of different oncogenes and the reversal of different tumor suppressor genes [64,65]. p53 is tumor suppressor gene and can be activated by different intrinsic and extrinsic factors, and activation of p53 is important for cell viability. Activation of p53 results in the induction of cellular differentiation and apoptosis. In addition, p53 can also modulate the activity of different downstream molecules. Most of theese downstream molecules are associated with the activity of different death receptors and molecules of mitochondria-dependent apoptotic pathways [66–68]. Following **1j** exposure, we observed an elevated expression level of p53 and down-regulation of a cell cycle regulatory molecule cyclin D1. The expression of different signaling intermediates due to exposure of **1j** and CDDP has been summarized schematically in Fig 9c.

From the above results and discussion, we got an indication that **1j** derivative might interfere with the progression of the cell cycle. Analyzing the cell cycle, we observed that the derivative potentially induces an arrest at G0/G1 checkpoint [69–71]. The effect of the bis-lawsone derivative on cell migration of the CCF-4 cells was found to be deleterious. Whereas untreated cells almost filled up the wounded space within a span of 24 hours, **1j** exposed cells were not able to migrate and fill up the wounded region. Further functional studies showed that **1j** exposure inhibited colony formation in CCF-4 cells. These results again indicated that this derivative may affect the microfilament network and anchorage dependent growth of the glioma cells [72].

To evaluate the anticancer potential of this novel bislawsone derivative (**1j**) further, the role of PI3K/ AKT/ mTOR pathway proteins were studied. Literature suggest that PI3K/ AKT/ mTOR pathway proteins are critical for the progession of cancer. Under normal circumstances these proteins facilitates cell proliferation, growth, differentiation, migration, and angiogenesis in all cell types [39]. From our experimental data, it can be said that **1j** derivative significantly downregulates the phosphorylations of PI3K, Akt, and mTOR compared to the control cells [73]. Combining, results from the present *in vitro* studies confirmed the proapoptotic nature of the compound, **1j**. In other words, **1j** significantly inhibited CCF-4 cell growth via the induction of apoptosis mediated by oxidative stress although it exhibits no significant toxicity to the normal cells.

In this connection, it is worth mentioning that the outcome of our experimental results is quite in accordance to the literature report indicating considerable pharmacological potential of a trifluoromethyl (-CF_3_) structural motif present in numerous bioactive compounds and functional materials [74–78]. It has also been observed that introduction of a trifluoromethyl group into organic compounds usually leads to improvement of their biological and physiological characteristics attributed to developing and/or improving unique physical and chemical properties (such as chemical and metabolic stability as well as bioavailability) in the trifluoromethyl-substituted derivatives by the incorporated trifluoromethyl group [78–82].

With all these properties, this **1j** molecule bearing a trifluoromethyl (-CF_3_) moiety can thus be considered as an anticarcinogenic agent and deserves further research with appropriately controlled in vivo experiments. Its fate to be used in the treatment of cancer finally depends on the clinical trial in future.
